# The rPRRSV-E2 strain exhibited a low level of potential risk for virulence reversion

**DOI:** 10.3389/fvets.2023.1128863

**Published:** 2023-03-07

**Authors:** Yifeng Jiang, Fei Gao, Liwei Li, Yanjun Zhou, Wu Tong, Lingxue Yu, Yujiao Zhang, Kuan Zhao, Haojie Zhu, Changlong Liu, Guoxin Li, Guangzhi Tong

**Affiliations:** ^1^Department of Swine Infectious Diseases, Shanghai Veterinary Research Institute, Chinese Academy of Agricultural Sciences, Shanghai, China; ^2^Jiangsu Co-Innovation Center for the Prevention and Control of Important Animal Infectious Disease and Zoonosis, Yangzhou University, Yangzhou, China

**Keywords:** rPRRSV-E2, virulence reversion, genetic stability, *in vivo* passage, CSFV E2

## Abstract

Porcine Reproductive and Respiratory Syndrome Virus (PRRSV) and Classical Swine Fever Virus (CSFV) are two important pathogens, which cause serious impact on swine industry worldwide. In our previous research, rPRRSV-E2, the recombinant PRRSV expressing CSFV E2 protein, could provide sufficient protection against the lethal challenge of highly pathogenic PRRSV and CSFV, and could maintained genetically stable *in vitro*. Here, to evaluate the virulence reversion potential risk, rPRRSV-E2 had been continuously passaged *in vivo*, the stability of E2 expression and virulence of the passage viruses were analyzed. The results showed that no clinical symptoms or pathological changes could be found in the inoculated groups, and there were no significant differences of viraemia among the test groups. Sequencing and IFA analysis showed that the coding gene of exogenous CSFV E2 protein existed in the passaged viruses without any sequence mutations, deletions or insertions, and could expressed steadily. It could be concluded that the foreign CSFV E2 gene in the genome of rPRRSV-E2 could be maintained genetically stable *in vivo*, and rPRRSV-E2 strain had relatively low level of potential risk for virulence reversion.

## 1. Introduction

Porcine reproductive and respiratory syndrome (PRRS) is characterized by late-term reproductive failure in sows and severe pneumonia in neonatal pigs (1–3). PRRSV is the causative pathogen of PRRS, which seriously affects the swine herds and causes huge economic losses to the pig industry worldwide (4–6). PRRSV is a positive-sense single-stranded RNA virus, belonging to the family *Arteriviridae* and genus *Arterivirus*. The 15-kb genome encoded at least 10 open reading frames (ORFs), which expressed at least 12 non-structural proteins (Nsps) and eight structural proteins (7–11). Although the level of bio-safety elevated since the ASF outbreak in 2018, PRRS is still one of the most significant threats to pig production in China (12–16).

Immunization is an effective measure to prevent and control PRRS, in which inactivated vaccines and subunit vaccines could not provide effective protection against PRRSV (17, 18). Live PRRS vaccines could stimulate cellular and humoral immune responses and provide respectable immune efficacy against heterologous PRRSV strains (19–23). In China, many live vaccines have been developed and used for PRRS control and contributed to the development of the pig industry (24, 25). However, the risk of virulence reversion of live vaccines has attracted extensive concerns and became one of the most important preclinical results in the innovation and development of a new vaccine (26–28).

Based on reverse genetic manipulation, several regions of the PRRSV genome could be used to insert foreign genes, to perform basic research or vaccine innovation (29–32). In the previous study, rPRRSV-E2-expressing CSFV E2 protein with the backbone of vaccine strain HuN4-F112 was engineered, and its genetic stability could be maintained *in vitro* for at least 25 consecutive cell passages (32). In this study, an *in vivo* trial was designed, and the potential risk for virulence reversion of rPRRSV-E2 was evaluated.

## 2. Materials and methods

### 2.1. Cells and viruses

African green monkey kidney cells (MARC-145 cells) were cultured in minimum essential medium (MEM; Invitrogen Corporation, Carlsbad, CA, USA) and supplemented with 10% fetal bovine serum (FBS; Life Technologies Inc., Gibco/Brl Division, Grand Island, NY, USA). It was maintained in MEM supplemented with 2% FBS at 37°C in a humidified atmosphere of 5% CO_2_, as described previously (33). The virus titer of recombinant strain rPRRSV-E2 was determined by performing standard median tissue culture infective dose (TCID_50_) assays in 96-well-plates. According to the Reed and Muench method (34), rPRRSV-E2 for experimental usage in this study is the fifth passage, and the virus titer is 10^6.0^TCID_50_/mL.

### 2.2. Immunofluorescence assays

The expression of PRRSV N protein and CSFV E2 protein in the experimental samples was determined by immunofluorescence assays (IFAs). MARC-145 cells grew to 70% confluence in six-well-plates and were infected with rPRRSV-E2 samples and mock controls, as reported. After 48 h of infection, cell monolayers were fixed with ice-cold methanol at room temperature for 10 min, blocked with 0.1% bovine serum albumin for 30 min, and incubated with 1:600 anti-PRRSV N (SR30A, Rural Technologies) or 1:1000 anti-E2 monoclonal antibody (prepared and preserved by our laboratory) at 37°C for 2 h. After washing with PBS five times, cells were incubated at 37°C for 1 h with Alexa Fluor 594-labeled goat anti-mouse IgG (H+L) (Invitrogen Corporation). After washing with PBS, fluorescence was visualized using an inverted fluorescence microscope (model IX741; Olympus Corporation, Tokyo, Japan), as previously mentioned (35).

### 2.3. Animal experiments

All pig experimental programs follow the guidelines for the Care and Use of Experimental Animals and are approved by the Ethics Committee of the Shanghai Veterinary Research Institute, Chinese Academy of Agricultural Sciences, with the number SHvri-pi-20190067. Fifty piglets at 30 days of age, which were PRRSV-free, CSFV-free, PCV2-free, and PRV-free, were purchased and bred. In the first generation, the recombinant vaccine strain rPRRSV-E2 was inoculated intramuscularly at a dose of 10^5.0^TCID_50_, and the serum samples with the highest viral load were selected to be inoculated with the second-generation to the fifth-generation virus at a dose of 2 mL. Five piglets per generation were inoculated with virus/serum samples (named R1–R5 groups, respectively, and each generation of pigs was one-to-one corresponded during the experiment). Another three piglets per generation served as the control group. When the virus was transmitted to the fifth generation, the serum samples of each pig in the fifth generation at the highest level of viremia were mixed, and then five piglets were inoculated. The original generation virus was inoculated at the same time, and the clinical manifestations were observed and recorded. After vaccination, the clinical manifestations of pigs were observed and recorded every day, such as feed intake, mental state, and abnormal conditions of the ears, eyelids, and body surfaces. After feeding, rectal temperature measurement was performed every morning until necropsy. Clinical anatomy observation: The experimental pigs were killed and necropsied on the 21st day after inoculation, and the pathological changes in inguinal lymph nodes, spleen, lung, kidney, and other tissues and organs were observed. Histopathological observation: The tissues of the inguinal lymph nodes, spleen, lung, and kidney of inoculated pigs were collected and their histopathological examination was carried out.

### 2.4. Analysis of viremia by real-time RT-qPCR

Blood samples and serum samples were collected 0, 7, 14, and 21 days after immunization, and the serum was split into four centrifuge tubes. RNA was extracted from one tube for real-time RT-qPCR from a blood sample using the QIAprep viral RNA Kit (Qiagen, Hilden, Germany) according to the manufacturer's instructions, as previously described. The other three test tubes were immediately frozen in a refrigerator at −70°C. RT-qPCR was used to detect and analyze the viremia of serum samples. Viral RNA was extracted from 140 μL of pig serum sample. Primers PNPF/PNPR and the NP probe were used for PRRSV RT-qPCR, and the sequences of primer pairs and probes are listed in Table 1.

**Table 1:** Oligonucleotides used for RT-PCR and qRT-PCR.

**Name^a^**	**Sequence^b^**	**Position^c^**	**Application**
HF11559	5′-TCATACATCCGAGTTCCTGTT-3′	11,559–11,579	PCR mutagenesis
HR13090	5′-GAAATATTGTCATGGCGAGGC-3′	13,070–13,090	PCR mutagenesis
HF11805	5′-TTTGAATCGGATACAGCGTATC-3′	11,805–11,826	Nucleotide sequencing
HR12100	5′-CCAAACAAAATGGCCAAAAATAT-3′	12,078–12,100	Nucleotide sequencing
PNPF	5′-CCCTAGTGAGCGGCAATTGT-3′	15,002–15,021	qRT-PCR primer (PRRSV)
PNPR	5′-TCCAGCGCCCTGATTGAA-3′	15,045–15,062	qRT-PCR primer (PRRSV)
NP-probe	FAM-TCTGTCGTCGATCCAGA-MGB	15,023–15,039	qRT-PCR probe (PRRSV)

^a^Abbreviations in primer names: F, forward PCR primer; R, reverse PCR primer.

^b^Italic lowercase represents sequences different from HuN4 (EF635006).

^c^Numbers in the primer names denote the nucleotide positions in the parental HuN4 (EF635006) and the CSFV Shimen strain (AF092448).

Re-isolation of virus: The first–fifth generation pig sera were taken and filtrated using a 0.22-μm filter, then inoculated with single-layer MARC-145 cells and adsorbed in an incubator at 37°C for 1 h. The inoculated serum was discarded, and DMEM culture solution containing 2% FBS was replaced and cultured at 37°C. The virus was collected when 60–70% CPE occurred, and RT-PCR amplification and the whole genome sequence determination were performed with PRRSV primers, of which sequences were listed in Table 1.

### 2.5. Statistical analysis

SPSS 14.0 software and GraphPad Prism 6.0 were used to perform all the data analysis and charting. Results: It was considered that there was a significant difference when the *p* < 0.05, and an extremely significant difference when the *p* < 0.01 or <0.001 (26).

### 2.6. Detection of the *in vivo* passage stability of exogenous gene

The rPRRSV-E2 strain isolated *in vivo* was passaged on MARC-145 cells to detect the stability of foreign genes in the process of virus passage. The stability of the marker gene CSFV E2 during the passage of the virus was determined. The MARC-145 cells infected by the virus were detected by IFA, and the expression of the CSFV E2 gene was observed.

## 3. Results

### 3.1. Clinical manifestations of experimental piglets showed normal conditions

**Figure 1:**
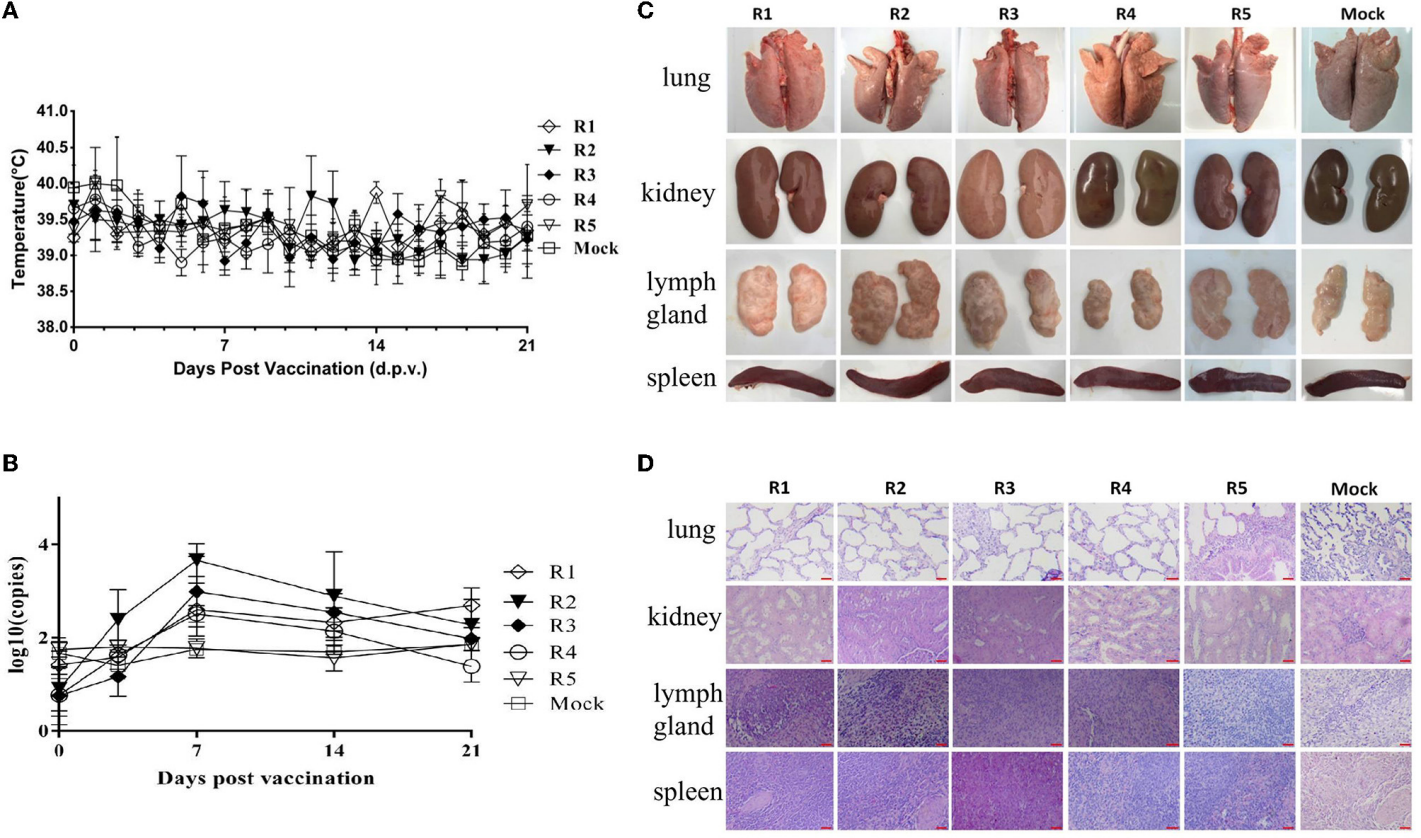
Safety identification of rPRRSV-E2 vaccination group and pig–pig *in vivo* serial passage sera inoculation group. The values of mock groups of each generation were indicated together. **(A)** Daily rectal temperature record of the rPRRSV-E2 inoculation group and the *in vivo* passage groups. **(B)** Viral RNA replication in serum samples detection for the rPRRSV-E2 inoculation group and the pig–pig *in vivo* generation sera inoculation group. Five piglets in each generation were inoculated with virus/serum samples, named R1–R5 groups, respectively, and each generation of pigs was one-to-one corresponded during the experiment. **(C)** Gross pathological changes of organs and tissues (spleen, lung, kidney, and lymph node) of rPRRSV-E2 inoculation group and *in vivo* passage sera inoculation group after necropsy. **(D)** Histopathological observation of organs and tissues (spleen, lung, kidney, and lymph node) of rPRRSV-E2 inoculation group and *in vivo* serial passage sera inoculation group after necropsy. Scale bar = 200 μm.

After the inoculation of each generation of virus/serum samples, the feed intake and mental state of the test pigs were normal, and there was no significant difference between the test and the mock control groups. There were no clinical symptoms such as anorexia, cough, diarrhea, congestion, or ulcer spots in the ears and body surface, no swelling of eyelids, and no obvious increase of eye secretion in pigs. After inoculation, all the pigs showed no symptoms of persistent hyperthermia (the body temperature was over 40.5°C, which was determined as persistent hyperthermia for 3 days). The temperature changes of the tested pigs are shown in Figure 1A. The serum samples were collected on the 7th, 14th, and 21st days after inoculation, which was used to determine the viral load in serum. The results showed that the virus load in pig serum of each group reached a peak in 7–14 days after inoculation (Figure 1B). However, the viremia level of R1–R5 groups gradually decreased, especially R5 group, their viremia levels were all below the negative value. In addition, viral shedding was also conducted to assess the risk. The results showed that the viral shedding levels were all below the negative threshold line (data not shown).

### 3.2. No obvious pathological changes were found in the clinical anatomy of piglets inoculated with rPRRSV-E2 at different generations

On the 21st day after inoculation, the experimental piglets were killed and necropsied. The dissection observation results showed that there were no visible pathological changes in the internal organs of each piglet of the rPRRSV-E2 *in vivo* passage group, and there was no significant difference compared with the blank control group (mock group) (Figure 1C). Histopathological examination was carried out on each group of organs, and the results showed that there was no obvious pathological damage to all kinds of parenchymal organs (spleen, lung, kidney, and lymph nodes) (Figure 1D).

### 3.3. Foreign CSFV E2 gene in the genome of rPRRSV-E2 was intact in the genetic stability analysis *in vitro* and *in vivo*

**Figure 2:**
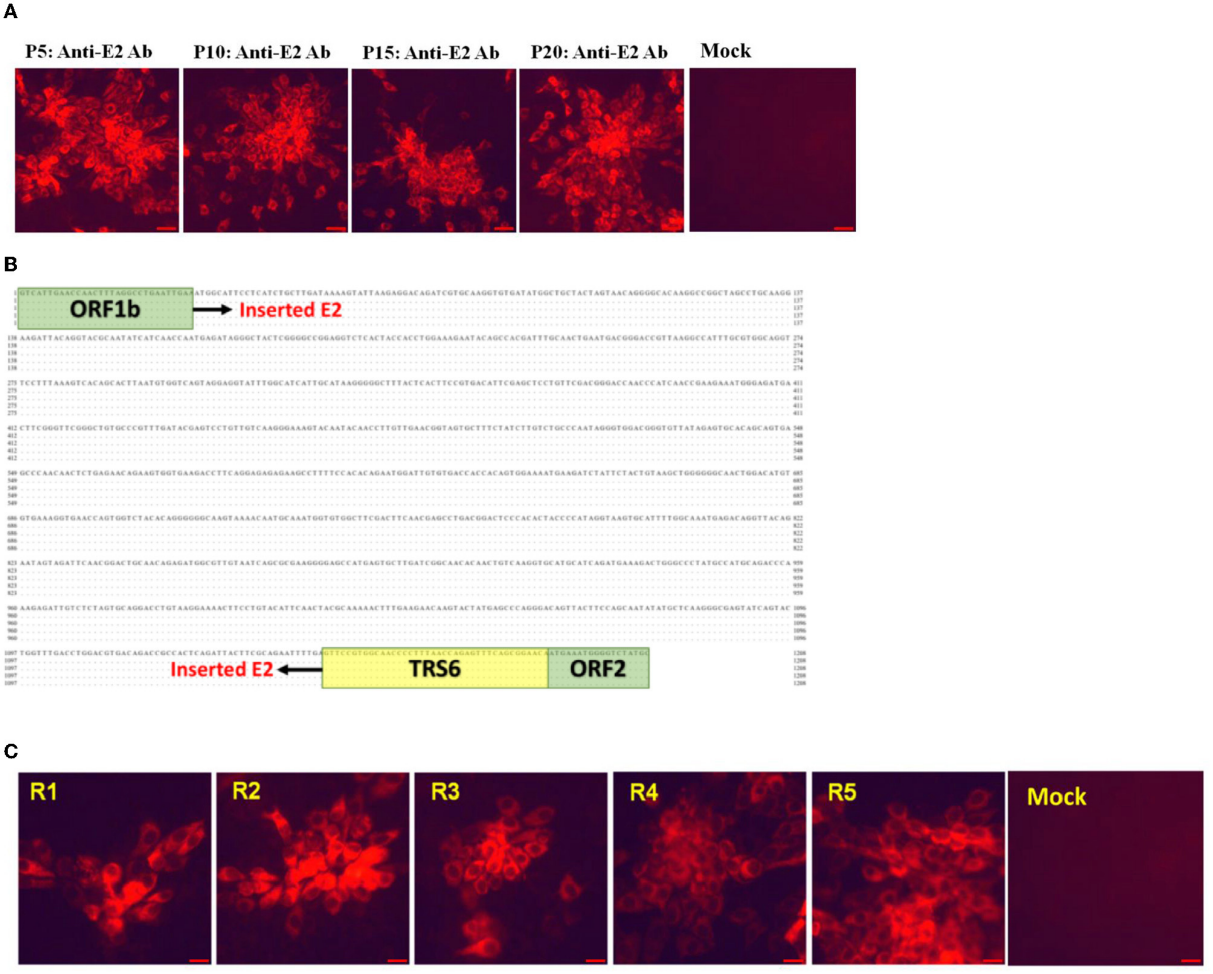
The genetic stability of foreign genes in rPRRSV-E2 *in vitro* and *in vivo*. **(A)** Indirect immunofluorescence results of CSFV E2 protein of *in vitro* generation of P5, P10, P15, and P20 viruses. **(B)** Sequence alignments of CSFV E2 protein insertion region of the isolated virus of various passages *in vivo*. **(C)** Indirect immunofluorescence assay of CSFV E2 protein expression of the isolated virus after passage *in vivo*. Scale bar = 20 μm.

The genetic stability analysis for rPRRSV-E2 was carried out in MARC-145 cells *in vitro*, and P5, P10, P15, and P20 viruses were sequenced. The sequencing results of the samples showed that the inserted CSFV E2 protein gene could exist stably in the viral genome, and no nucleotide variation occurred. The results of IFA showed that the E2 gene of CSFV could be expressed normally (Figure 2A). The whole genome of each generation of the virus during *in vivo* passage was sequenced. The results of stability analysis of the CSFV E2 gene showed that compared with its parental strain, the virus isolated *in vivo* (R1–R5) had no nucleotide mutation in the E2 insertion region (Figure 2B). Only a few base mutations were scattered in the whole genome. Neither base insertion nor deletion was found. IFA for CSFV E2 protein of the isolated virus showed that E2 protein was stably and abundantly expressed (Figure 2C).

### 3.4. High degree of safety could be proved in the comparative evaluation between *in vivo* passage serum mixture inoculated group and the original virus inoculated group

**Figure 3:**
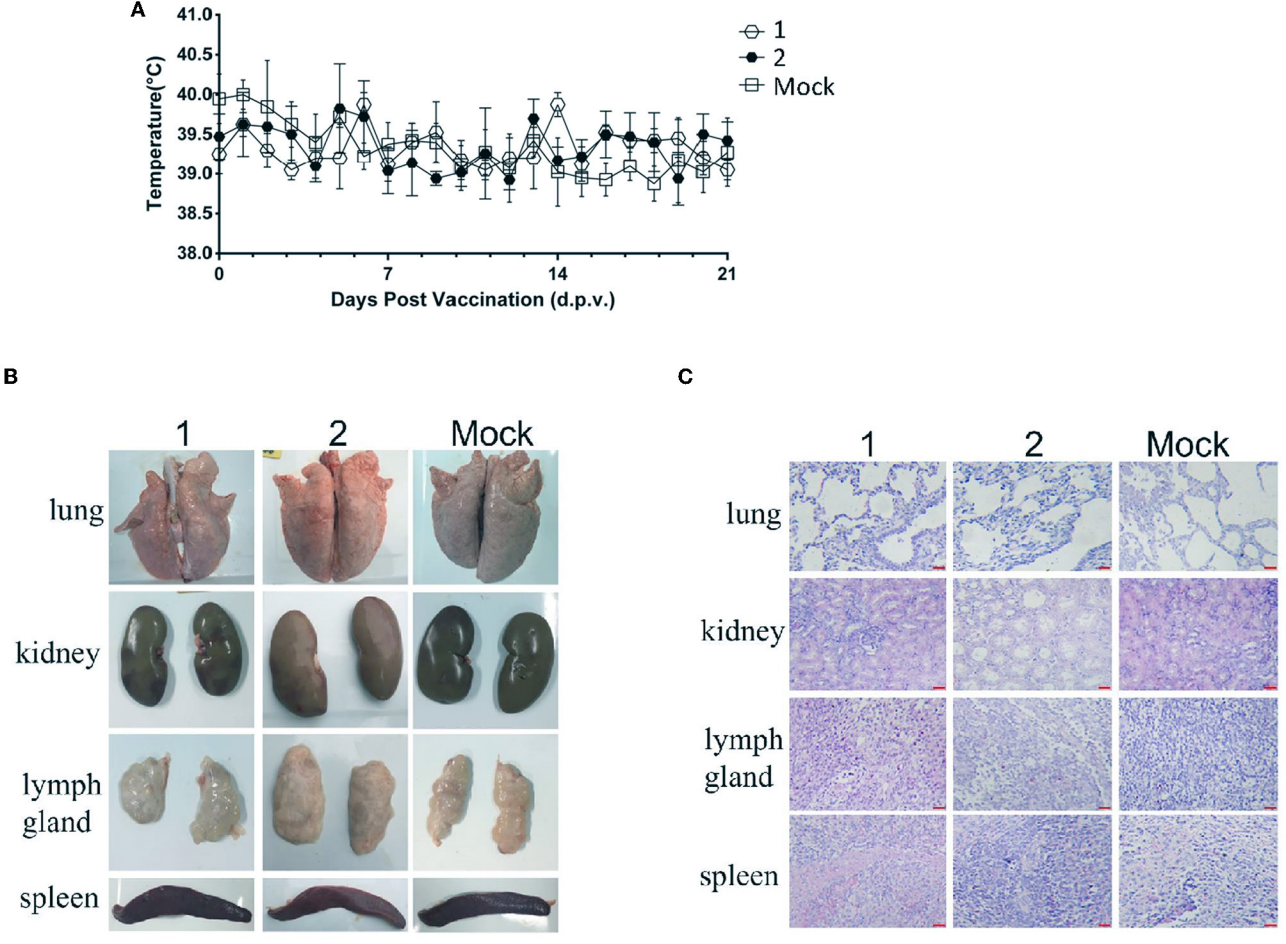
Safety comparison test between *in vivo* passage serum mixture inoculated group and original virus inoculated group. The changes in rectal temperature of pigs in each group after inoculation, autopsy results, and histopathological observation. **(A)** Daily rectal temperature record of rPRRSV-E2 inoculation group compared with *in vivo* passage sera inoculation group. **(B)** Gross pathological changes of organs and tissues (spleen, lung, kidney, and lymph node) of rPRRSV-E2 inoculation group (1) and *in vivo* passage mixed-sera inoculation group (2) after necropsy. **(C)** Histopathological observation of organs and tissues (spleen, lung, kidney, and lymph node) of rPRRSV-E2 inoculation group (1) and *in vivo* passage mixed sera inoculation group (2) after necropsy. Scale bar = 200 μm.

After inoculation, there was no redness and inflammation in the inoculated parts of piglets in the fifth-generation virus inoculation group and the original virus inoculation group. The feed intake and mental state of the experimental piglets inoculated with the fifth-generation virus were normal, and there was no significant difference between the blank control group and the fifth-generation virus inoculation group. There was no congestion or ulcer spots on the ears and body surfaces of inoculated piglets, no swelling of eyelids, and no obvious increase in the secretions of eyes. All the piglets did not show symptoms of persistent hyperthermia after inoculation (Figure 3A). Autopsy results showed that no visible pathological changes were found in the parenchymal organs and the lymph nodes of the piglets in each group (Figure 3B). Histopathological observation showed that there was no obvious pathological damage to the spleen, lung, kidney, lymph nodes, and other parenchymal organs of the pigs in each group (Figure 3C).

## 4. Discussion

Since it emerged in the late 1980's, many kinds of vaccines have been developed for PRRS control, including inactive, subunit, and attenuated live vaccines. However, only live vaccines showed sufficient immune protection and are used in major pig breeding areas all over the world (17, 18, 20, 23). Although PRRS spreads and evolves rapidly, immunization still plays a very positive role in the prevention and control of PRRS (36, 37). On the other hand, live vaccines still have many defects to be improved, including the risk of virulence reversion, which has been reported by many studies and has become one of our foremost concerns of PRRSV vaccine innovation and development (26–28).

In this study, the recombinant PRRSV live vector vaccine rPRRSV-E2 strain was concerned. The E2 gene, an important protective antigen of CSFV, was inserted into the intergenic region of ORF1b and ORF2 of PRRSV by reverse genetic manipulation. Previous studies had proved that the vaccine could provide good immune protection for both HP-PRRSV and CSFV (32). The potential risk of virulence reversion for rPRRSV-E2 was assessed in this study. The results of this study showed that during the observation period, piglets inoculated P1–P5 rPRRSV-E2/serum did not show any PRRS-related clinical symptoms or visible pathological lesions in the lungs, kidneys, lymph nodes, and spleens, which demonstrated the safety of the passaged viruses and indicated that rPRRSV-E2 had no virulence reversion of *in vivo*, at least in these five passage processes. The stability of the PRRSV genome was very important for virulence. In the evolution history of PRRSV, the virulence change is always accompanied by great changes in the genome (38–40). The results showed that the coding gene of exogenous CSFV E2 protein existed in the passage viruses, without any sequence mutations, deletions, or insertions, and it could be stably expressed. Combined with the previous study, full-length sequencing of P5, P10, P15, P20, and P25 rPRRSV-E2 virus stocks of MARC-145 cells showed that there were no mutations in the region where foreign genes were expressed, and they remained stable at least for 25 consecutive cell passages (32). The genome of rPRRSV-E2 was stable *in vivo* and *in vitro*, and this will reduce the possibility of virulence reversion.

For live PRRSV vaccine, most studies consider that there are high risks of virulence reversion. The causes for this phenomenon should be considered from the following aspects: 1. Genetic stability of gene sequences; 2. Horizontal transmission capability of vaccine; and 3. The level and duration of viremia. In this study, the potential risk of virulence reversion of the rPRRSV-E2 strain was evaluated, and the results showed that the risk was relatively low. First, the genetic stability test *in vitro* and *in vivo* confirmed that the virus sequences of the rPRRSV-E2 strain, especially the sequences of foreign genes, were stable. In addition, rPRRSV-E2 had passed the transgenic safety evaluation. The experiment of horizontal transmission confirmed that the pigs immunized with the rPRRSV-E2 strain were kept in the same house as normal piglets and could not infect other non-immunized piglets (data not shown). There is no ability and risk of horizontal transmission. Moreover, compared with the parental vaccine strain vHuN4-F112, the rPRRSV-E2 strain has a shorter duration and a lower viremia level, because the insertion of the exogenous CSFV E2 gene makes it further weakened and has higher safety characteristics. In addition, previous studies also found that the virus shedding and virus load levels of rPRRSV-E2 in tissues were low. All these causes and factors determined that rPRRSV-E2 showed little potential risk in the assessment of virulence reversion. Furthermore, it is worth noting that the reverse genetic manipulation platform for full-length infectious cDNA clone of pHuN4-F112 is stable and convenient, and it exhibits a strong ability to accommodate foreign genes. Thus, far, we have found that multiple sites and foreign genes can be stably inserted based on this platform, and the maximum foreign gene size can reach 2,100 bp. Multiple foreign genes can be simultaneously and stably expressed. Therefore, stability is essential for PRRSV to be used as a platform for the development to develop new genetic engineering live vaccines. This may also be one of the reasons for the good performance of rPRRSV-E2 in the risk assessment of virulence reversion.

Virulence reversion is determined by many factors, including the frequency, correct use of the vaccine, selection of generation to be used as vaccines, and the characteristic of the candidate vaccine strain. This vaccine strain, which was reported with virulence reversion, mainly used the virus that has been passaged on MARC-145 cells for <100 generations (26–28). rPRRSV-E2 was generated by the full-length infectious clone of the vaccine strain HuN4-F112, which was made from the serial passage of wild-type HuN4 on MARC-145 cells for 112 generations (24, 41). HuN4-F112 was proved to be safe, and the genome was stable from generations 112–130, which increases the safety of rPRRSV-E2. We already exhibited the stability of the rPRRSV-E2 genome and the virulence of *in vivo* passage viruses. A previous study also demonstrated that there was no virus shedding after rPRRSV-E2 inoculation and no horizontal transmission among the co-inhabited piglets, which also indicated the safety of rPRRSV-E2 (32). Virulence reversion was determined by many factors, although the safety of rPRRSV-E2 is demonstrated, the scientific and rational usage of the vaccine still needs more attention, and the PRRS vaccine needs to be upgraded continuously.

In conclusion, rPRRSV-E2 is a candidate vaccine strain that is safe for immunization. The genome was observed to be stable *in vivo* and *in vitro*, and the level of potential risk for virulence reversion was relatively quite low.

## Data availability statement

The original contributions presented in the study are included in the article/supplementary material, further inquiries can be directed to the corresponding authors.

## Ethics statement

The animal study was reviewed and approved by Ethics Committee of the Shanghai Veterinary Research Institute, Chinese Academy of Agricultural Sciences.

## Author contributions

This study was conceived and designed by GL and GT. YJ and FG performed the experiments and prepared the figures. FG, LL, and YZho wrote the main manuscript text. WT and LY analyzed the data. YZha, KZ, and HZ prepared the manuscript. GT made constructive comments on the experiments. All authors participated in the experiments, contributed to the article, and approved the submitted version.
